# Physical punishment of children by US parents: moving beyond debate to promote children’s health and well-being

**DOI:** 10.1186/s41155-018-0096-x

**Published:** 2018-07-03

**Authors:** Cindy Miller-Perrin, Robin Perrin

**Affiliations:** Social Science Division, Pepperdine University, 24255 Pacific Coast Highway, Malibu, CA 90263 USA

**Keywords:** Physical punishment, Spanking, Cultural norms, Human rights

## Abstract

Physical punishment remains a common practice in the USA despite significant empirical evidence of its potential harm and ineffectiveness, arguments that its use violates children’s human rights, and professional recommendations against its use. The purpose of the current paper is to offer explanations as to why, in the face of a worldwide movement to protect children from violence, the USA continues to support physical punishment of children. The paper also summarizes the various debates engaged in by experts that stem from these explanations for physical punishment and argue that the time has come to move beyond these debates and eliminate the physical punishment of children. We offer suggestions for changing attitudes and practices related to physical punishment of children in order to promote their health and well-being. We conclude by suggesting that the burden of proof in debates about physical punishment, which has typically fallen upon those who argue children should never be physically punished, should shift to those who continue to promote its use despite evidence of its harm and ineffectiveness.

## Introduction

The physical punishment of children, defined as “any punishment in which physical force is used and intended to cause some degree of pain or discomfort” (United Nations, Committee on the Rights of the Child [UNCRC], 2007, p. 4) is a worldwide issue that has attracted considerable attention in recent years. Attitudes and practices regarding physical punishment and its most common form, spanking with an open hand, have dramatically changed around the world. The United Nations Committee on the Rights of the Child, which was adopted in 1989, states in Article 19 that member nations should “take all appropriate legislative, administrative, social and educational measures to protect the child from all forms of physical or mental violence.” The Committee stressed that all physical punishment of children, including that in homes, should be eliminated through “legislative, administrative, social and educational measures” (Committee on the Rights of the Child, 2006, para. 18). Currently, 196 countries are party to the treaty, including every member of the United Nations except the USA. Parental use of physical punishment has also been banned in more than 50 countries to date (Global Initiative to End All Corporal Punishment of Children, 2017). These trends suggest a decrease in attitudes and practices supportive of physical punishment of children and are no doubt due to a number of factors, including empirical research questioning its use, as well as international legislation suggesting that physical punishment violates the human rights of children (Gershoff & Grogan-Kaylor, 2016; UNCRC, 2007).

This worldwide movement to end the physical punishment of children should not lead us to conclude, however, that the problem is solved. In many of the 196 nations who are party to the United Nations Convention on the Rights of the Child, physical punishment of children remains common (Lansford et al., 2017). For example, in South Korea, which ratified the United Nations Convention on the Rights of the Child in 1991, physical punishment of children remains common. A national study on the prevalence rate of violence toward children led the researchers to conclude, “Korean society needs to lower significantly its tolerance level for all forms of violence,” and to “raise awareness regarding the ineffectiveness and damaging effects of corporal punishment” (Ahn et al., 2017). In the USA, physical punishment, while declining (see Straus, Douglas, & Medeiros, 2014), also continues to be viewed largely positively and is commonly practiced.

## Review

The purpose of the current paper is to offer explanations as to why, in the face of worldwide movement to protect children from violence, physical punishment against children remains normative in much of the world. We focus on the USA because the use of physical punishment by parents is very common in the USA, but many of the issues we highlight are relevant to other countries. We also summarize the various debates about physical punishment and argue that the time has come to move beyond these debates to eliminate the physical punishment of children. We offer suggestions for changing attitudes and practices related to physical punishment of children in order to promote their health and well-being. Finally, we conclude by suggesting that the burden of proof in debates about physical punishment, which has typically fallen upon those who argue children should never be physically punished, should shift to those who continue to promote its use despite evidence of its harm and ineffectiveness.

### Support for physical punishment in the USA

Although US federal law does not provide a definition of physical punishment, its legality is typically stated in the form of a provision that gives parents immunity from prosecution for child physical abuse if the actions are deemed to represent “reasonable force” when used for the purposes of correction and control (Straus, 2010). Unreasonable force, or child physical abuse, is defined by actions that result in “serious physical or emotional harm” (Child Abuse Prevention and Treatment Reauthorization Act, 2010). In all 50 states, physical punishment by parents—that is, acts that may cause physical pain but that do not cause harm or injury—is legal.

Attitudinal and behavioral surveys of the US population generally support the legal status of physical punishment. Three fourths of adults agree or strongly agree with the statement, “It is sometimes necessary to discipline a child with a good, hard spanking” (Smith, Hout, Marsden, & Kim, 2015), and behavior surveys estimate that 80% of parents have spanked their child at some point during childrearing (Gershoff, Lansford, Sexton, Davis-Kean, & Sameroff, 2012).

#### Explaining support for physical punishment in the USA

Some argue that the fundamental parental right to control the upbringing of their own child is protected by the Fourteenth Amendment (Pagliocca, Melton, Lyons Jr, & Weisz, 2002). While this interpretation is a subject of considerable legal debate (Shulman, 2014), the fact remains that the dual concepts of “parental freedom” and “parental rights” are strongly embedded in the US culture (Lane, 1998). Parental rights and freedoms, furthermore, continue to be upheld by US courts and judges, even under conditions involving harsh physical punishment (Moya-Smith, 2013; Schworm, 2015). For example, in a 2013 California case, in which a mother who hit her daughter five or six times with a wooden spoon, resulting in bruises, the judge on the case concluded that the local department of social services violated the mother’s right to impose reasonable discipline upon her child (Moya-Smith, 2013).

The high rate of adherence to Christianity in the USA, especially compared to Europe, also contributes to high rates of support for corporal punishment. Conservative Protestants, in particular, are likely to believe that parents have a right and a responsibility to impose physical discipline. Several Old Testament passages, primarily from the book of Proverbs, have often been interpreted by Conservative Christians to mean that a parent who spares the rod, spoils the child (Ellison & Sherkat, 1993; Perrin, Miller-Perrin, & Song, 2017).

The view that physical punishment is harmless is also reflected, to some degree, in the attitudes of professionals. In a survey of medical center staff conducted by Gershoff et al. (2016), less than half of medical center staff agreed that spanking is harmful to children. In a recent survey of psychologists, approximately one-third were either unsure or disagreed that spanking is harmful to children (Miller-Perrin C. & Rush, R: Attitudes, knowledge, practices, and ethical beliefs of psychologists related to spanking: A survey of American Psychological Association division members, submitted).

A final reason that cultural beliefs persist is reflected in parental attitudes and behaviors toward child discipline. Most people are spanked in the USA, and parents tend to parent the way they were parented (Graziano & Namaste, 1990). In addition, some believe that because they were spanked and “turned out ok,” physical discipline is effective and harmless (Kish & Newcombe, 2015). Others believe that children who are spanked are disciplined and respect authority, while those who are not are uncontrolled and disrespectful (Benjet & Kazdin, 2003). Finally, many US adults equate discipline with physical punishment, as if spanking were the only way to discipline a child (Knox, 2010).

#### Hitting is rarely viewed as a violent act

Some object to the term “hitting,” when describing the physical punishment of children arguing that it is biased, or too harsh, or has an overly negative connotation. However, as Straus (2010) notes, the word “hit” is no more inherently biased or harsh than any other label that might be used to describe “spanking” (e.g., “swat,” “paddle,” “whack,” “beat,” “whip”). Most US child advocates, as well as those in other countries, actually expand the term “hitting” one step further, arguing that hitting is a form of violent behavior. Violence is “an act carried out with the intention of, or an act perceived as having the intention of, physically hurting another person” (Steinmetz, 1987). Physical punishment, including spanking, is, by definition, a violent act. It is intended to hurt—cause pain—to a child. Physical punishment of children may be “subabusive violence” (Graziano & Namaste, 1990), it may be “acceptable violence,” but it is still a form of violence, no matter what we call it.

In the USA, it is not socially acceptable for a husband to hit his wife, or for a wife to hit her husband. Children on a playground are not allowed to hit. Even dogs, many would argue, should never be hit. Children, however, are the only members in US society for whom hitting is deemed acceptable. One reason why hitting children might be deemed permissible could be the reluctance of adults to view physical punishment as a form of violence. As Bussmann and colleagues have noted, “parents are not aware of the contradiction between their attitude toward nonviolent childrearing and their own use of corporal punishment, simply because they do not define what they do as violence” (Bussmann, Erthal, & Schroth, 2011).

### Moving beyond debates about physical punishment of children

Debates about parental use of physical punishment have been ongoing in the USA for decades. Calls to “move beyond” the research, or to “end the debate,” have become commonplace (e.g., Durrant & Ensom, 2017; MacMillan & Mikton, 2017). Three questions, it seems, sit at the center of these debates. Is physical punishment of children a Human Rights Issue? Is physical punishment effective? Is physical punishment harmful?

#### Physical punishment is a human rights issue

A growing number of international bodies have declared that physical punishment is a violation of a child’s human rights, including six multilateral human rights documents and treaties (Bitensky, 2006; Gershoff & Bitensky, 2007). Most significantly, as discussed above, the United Nations, as articulated in its Convention on the Rights of the Child (United Nations Convention on the Rights of the Child, 1989), states that physical punishment is a form of “legalized violence against children” (Committee on the Rights of the Child, 2006, para. 18) that is prohibited by Article 19 of the CRC’s prohibition on “all forms of physical or mental violence” (United Nations Convention on the Rights of the Child, 1989, Article 19, para. 1).

The USA signed the treaty in 1995 but has never ratified it. This means, essentially, that the USA has endorsed the general principles outlined in the treaty but is unwilling to be legally bound by the treaty’s mandates. This unwillingness to ratify illustrates the continuing tensions between those who affirm and promote “children’s rights” and those who affirm and promote “parental rights.” Criticism of the treaty has come, in large part, from the political and religious right, which has expressed concerns that the treaty would infringe on the rights of parents (Ruck, Keating, Saewyc, Earls, & Ben-Arieh, 2016).

It is important to note, however, that physical punishment of children is *not* permitted in most US settings outside the home. It is typically not permitted in child care settings, schools, residential treatment facilities, or juvenile detention facilities (Bitensky, 2006). While much has been made of the fact that physical punishment is allowed in schools in *some* states, it is important to note that it is illegal in 31 states (Gershoff & Font, 2016). As a society, we appear uneasy about the value of physical punishment of children by individuals who do not hold the role of parent, yet we defer to the parent’s “right” to use physical discipline in the home. The United Nations Secretary General’s Study on Violence Against Children has called for explicit prohibition of physical punishment and other forms of “cruel or degrading punishment” in *all* settings, including the home (United Nations, 2006). Furthermore, the study report stated that “No violence against children is justifiable; all violence against children is preventable. The study marks the end of adults’ justification of violence against children, whether accepted as tradition or disguised as discipline” (United Nations, 2006, p. 17).

If, in the USA, we hope to end the debate on whether physical punishment of children is a human rights violation, the continuing ambiguity with the United Nations Convention on the Rights of the Child must be reconciled. Because ratification of a treaty in the USA requires a two-thirds majority in Congress, it is difficult to imagine ratification any time soon. However, we can still reconcile our continuing ambiguity by acknowledging a fundamental truth: there is no such thing as a “parental right.” Humans—which includes both parents and children—have rights. And all human beings, including children, have a right *not* to be hit.

#### Physical punishment is ineffective and harmful

Decades of research has yielded more than 500 studies examining the impact of physical punishment on children (Gershoff & Grogan-Kaylor, 2016). Within the past 15 years, several meta-analyses have attempted to synthesize this body of research. In a highly publicized meta-analysis, Gershoff (2002) concluded that physical punishment is not only ineffective, but also harmful. Larzelere and Kuhn then conducted a meta-analysis in 2005 and concluded that physical punishment may be effective if used conditionally, such as an open-handed swat on the buttocks with 2- to 6-year-olds when other forms of discipline have been unsuccessful (Larzelere & Kuhn, 2005). Ferguson (2013), who also conducted a meta-analysis on the long-term impact of physical punishment, concluded that the negative effects, while statistically significant, are “trivial to small.” Researchers, he argues, “should take greater care not to exaggerate the magnitude and conclusiveness of the negative consequences.” Exaggerations “might easily backfire, decreasing the credibility of scholarly statements on parenting research overall” (Ferguson, 2013, p. 204).In a definitive meta-analysis examining 50 years of research on outcomes associated with spanking, Gershoff and Grogan-Kaylor (2016) attempted to address two issues about the quality and interpretation of the spanking research, in particular. The first issue has to do with the potential confound between potentially abusive physical punishment and spanking. The second issue has to do with the assertion that physical punishment, including spanking, has only been linked to harmful outcomes in methodologically weak studies. Gershoff and Grogan-Kaylor analyzed 75 studies in the context of these two questions and found no evidence that nonabusive spanking is more effective than other disciplinary techniques at securing children’s immediate or long-term compliance. Indeed, they found that spanking was associated with increased risk of 13 harmful outcomes. Spanking and hitting children was a risk factor for adverse effects on such important outcomes as children’s aggressive behavior, mental health, and relationships with parents. Of all of the outcomes studied, physical abuse victimization was linked most strongly with spanking. Additionally, these researchers found no evidence that spanking is only associated with harmful outcomes in methodologically weak studies. Although not every incidence of spanking results in negative outcomes, the preponderance of evidence clearly suggests that it is ineffective and a risk factor for negative developmental outcomes.

Critics of this research argue, not unreasonably, that almost all of the research on harm and ineffectiveness is non-experimental, making causal connections difficult to establish. This research, they argue, cannot *prove* a causal link between spanking and harm. However, as defenders of the research remind us, much of the correlational evidence comes from studies that are statistically rigorous and use multi-variate models that attempt to control for extraneous variables. These studies have consistently documented that physical punishment increases the risk that children will experience harm or develop behavior problems, suggesting a causal pathway from parental physical punishment to negative developmental outcomes (Gershoff & Grogan-Kaylor, 2016; Gershoff, Sattler, & Ansari, 2017). Plus, defenders of the research argue it is neither feasible nor ethical, to randomly assign parents and children into experimental and control groups. As a result, pure experimental evidence on this issue will never be available. It is important to note that any number of public health and safety policy issues rest not on experimental but rather correlational evidence (e.g., see American Psychological Association, 2018).

#### Professional best practice standards suggest no physical punishment of children

In large part, the underlying cultural support for spanking and debates over the empirical evidence have undermined advocacy efforts to end physical punishment of children. As a result, advocacy and policy organizations have sometimes been reluctant to condemn, or even recommend against, spanking. For example, despite the American Psychological Association’s (APA) Code of Ethics, which obligates psychologists to “do no harm,” to safeguard vulnerable populations, and to protect individuals’ human rights (American Psychological Association, 2010), the organization has resisted the approval of a resolution opposing the use of physical punishment of children by parents. This is true despite the fact that the APA passed a resolution in 1975 opposing physical punishment in schools and other institutions based on far less empirical evidence than is currently available.

Of course, given the overwhelming cultural acceptance of physical punishment in the USA, such resistance on the part of professional organizations is in many ways predictable. As Straus (2010) notes, it took several years of bitter debate before the American Academy of Pediatrics was able to adopt a policy advising parents not to use physical punishment. According to Straus, the publication of this document required a compromise in wording to exclude hitting a child with an open hand, and the document carefully avoids suggesting that parents should *never* spank (American Academy of Pediatrics, 1998).

Professional attitudes, however, are changing rapidly. A survey of psychologists conducted 40 years ago found that the majority (51%) of those who worked with parents recommended spanking as a discipline technique (Anderson & Anderson, 1976). In contrast, 65% of a sample of APA members in 1990 reported that they had *never* recommended that parents spank their children (Rae & Worchel, 1991). By 2000, 70% of a sample of psychologists stated that they would never recommend that a parent spank a child (Schenck, Lyman, & Bodin, 2000). In a survey published in 2016, 86% of practicing psychologists indicated that they “never” advise a parent to spank a child with a hand (Miller-Perrin C. & Rush, R: Attitudes, knowledge, practices, and ethical beliefs of psychologists related to spanking: A survey of American Psychological Association division members, submitted).

Other recent surveys suggest that the majority of mental health professionals, as well as other professionals such as physicians and child welfare personnel, do not support the use of physical punishment. In one study, three-fourths of professionals believed that spanking is a “bad disciplinary technique” (Taylor, Fleckman, & Lee, 2017). In Gershoff et al.’s (2016) study of medical center staff, only about 15% agreed that “sometimes the only way to get a child to behave is with a spank.” Similarly, Miller-Perrin and Rush (Miller-Perrin C. & Rush, R: Attitudes, knowledge, practices, and ethical beliefs of psychologists related to spanking: A survey of American Psychological Association division members, submitted) found that only 15% of psychologists agreed that “sometimes the only way to get a child to behave is with a spank.” Furthermore, when this group of psychologists was asked whether spanking is a more appropriate part of parenting for some groups than others, such as some ethnic and religious groups, only 32% and 26%, respectively, agreed or strongly agreed. Many professionals are also changing their views about whether spanking represents an important ethical issue. In a survey of psychologists conducted in 2000, just one third of psychologists believed it was unethical to recommend that a parent use spanking. By 2016, that number had risen to 76% (Miller-Perrin C. & Rush, R: Attitudes, knowledge, practices, and ethical beliefs of psychologists related to spanking: A survey of American Psychological Association division members, submitted). According to these studies, a majority of professionals who work with children believe that spanking should be avoided.

The tide of professional opinion has clearly shifted. Many US professional organizations dedicated to the welfare of children and families have issued statements in recent years recommending that parents refrain from using physical punishment with their children. These organizations include the American Academy of Child and Adolescent Psychiatry (2012), the National Association of Pediatric Nurse Practitioners (2011), and the American Professional Society on the Abuse of Children (2016), among others. The American Psychological Association may be next. Approximately 60% of APA division members believe that the APA should adopt both a policy opposing any use of spanking or physical punishment by parents/caregivers and a policy stating that its member should never recommend spanking or physical punishment (Miller-Perrin C. & Rush, R: Attitudes, knowledge, practices, and ethical beliefs of psychologists related to spanking: A survey of American Psychological Association division members, submitted). This represents a significant shift in opinion from 2000 when approximately one-third of psychologists thought that APA should adopt such policies.

## Conclusion

Over the last 50 years, the physical punishment of children has attracted considerable empirical attention from social scientists. This research suggests, overwhelmingly, that spanking does more harm than good (see Ferguson, 2013; Gershoff & Grogan-Kaylor, 2016; Gershoff, Sattler, & Ansari, 2017; Gromoske & Maguire-Jack, 2012). Physical punishment has also been acknowledged internationally as a violation of human rights (United Nations, 2015). In addition, many professional health organizations have called on parents to abandon physical punishment as a child disciplinary practice (e.g., American Academy of Child and Adolescent Psychiatry, 2012; American Academy of Pediatrics, 1998). Professional experts who work with children and families also overwhelmingly believe that spanking is a bad disciplinary technique, is harmful to children, is unethical to recommend to a parent, and would never advise parents to spank their child (Miller-Perrin C. & Rush, R: Attitudes, knowledge, practices, and ethical beliefs of psychologists related to spanking: A survey of American Psychological Association division members, submitted).

The time has come to move beyond contentious debates. The verdict is in: physical punishment does more harm than good and parents and professionals should act according to that which promotes children’s rights to health and well-being. The way forward will necessarily involve a number of approaches.

- First, US culture can no longer hide behind euphemisms for violence. Physical punishment is hitting children and a form of violence against them.
- Second, parents should be educated about the empirical research on hitting children and the merits and techniques of non-violent parenting. Several promising intervention strategies to reduce parents’ use of physical punishment have been evaluated and shown to be effective (Gershoff, Lee, & Durrant, 2017). One such program is ACT, created by the American Psychological Association (www.apa.org/act/). The ACT program, which teaches parents and caregivers positive and non-violent parenting, has met with considerable success with research indicating that parents who participate in such programs report using physical punishment significantly less often (e.g., spanking and hitting with an object) and increasing their use of positive parenting practices (e.g., nurturing behavior) compared to parents in control groups (Knox, Burkhart, & Cromly, 2013; Knox, Burkhart, & Howe, 2011; Portwood, Lambert, Abrams, & Nelson, 2011).
- Third, professionals need to be educated about the physical punishment research on outcomes and how to talk with parents about never using physical punishment. Professionals that should be targeted include all those who work with children and families such as health care providers, psychologists, child welfare professionals, teachers, child care providers, and religious leaders. Educating professionals on the physical punishment research has been shown to reduce positive attitudes toward it (Hornor et al., 2015) and increase the likelihood that medical staff would intervene if they observed physical punishment (Gershoff & Font, 2016).
- Fourth, public health campaigns could be helpful in educating the general public about physical punishment, including media campaigns similar to those used to help prevent child abuse (public service announcements, billboard campaigns, etc.). Evidence suggests that such media campaigns can be effective at increasing awareness about the harm associated with physical punishment (Bussmann et al., 2011).
- Fifth, increased advocacy for laws against spanking is needed. Research in countries like Sweden, which in 1979 criminalized spanking, suggests that criminalization led to large reductions in the use of physical punishment, especially severe physical punishment (Durrant, 2000; Durrant, Rose-Krasnor, & Broberg, 2003). Zolotor and Puzia (2010) studied 24 countries that have passed legislative bans and likewise concluded that both general support of physical punishment and actual use of physical punishment declined following the enactment of the ban. Such bans are effective in and of themselves in decreasing physical punishment but may be enhanced by campaigns to promote their awareness and to educate parents about alternative forms of discipline (Lansford et al., 2017).
- Sixth, we must acknowledge the important role of unique cultural beliefs—such as religious beliefs—that create and maintain positive attitudes toward physical punishment of children. Interventions that are sensitive and respectful toward parents’ cultural views and values while attempting to change attitudes can be effective (Miller-Perrin & Perrin, 2017; Perrin et al., 2017).
- Finally, researchers should continue to design studies that make causal assertions more reasonable (Gershoff & Grogan-Kaylor, 2016; MacMillan & Mikton, 2017). In addition, although the burden of proof in debates about physical punishment has typically fallen upon those who argue children should never be physically punished, perhaps it is time for a shift in perspective. Advocates opposed to physical punishment of children have been asked, essentially, to provide empirical evidence that spanking does more harm than good. Perhaps, it is time to place the burden of proof on the defenders of physical punishment and ask the *defenders* of physical punishment to provide empirical support that spanking does more good than harm.

## References

[CR1] Ahn J, Lee BJ, Kahng SK, Kim HL, Hwang OK, Lee EJ (2017). Estimating the prevalence rate of child physical and psychological maltreatment in South Korea. Child Indicators Research.

[CR2] American Academy of Child and Adolescent Psychiatry. (2012). Policy statement on corporal punishment. Retrieved from: https://www.aacap.org/aacap/policy_statements/2012/Policy_Statement_on_Corporal_Punishment.aspx

[CR3] American Academy of Pediatrics, Committee on Psychosocial Aspects of Child and Family Health (1998). Guidance for effective discipline. Pediatrics.

[CR4] American Professional Society on the Abuse of Children. (2016). APSAC Position Statement on Corporal Punishment of Children. Retrieved from: https://www.apsac.org/

[CR5] American Psychological Association (2010). Ethical principles of psychologists and code of conduct.

[CR6] American Psychological Association. (2018). Resolution on Male Violence Against Women. Retrieved from https://www.apa.org/about/policy/male-violence.aspx

[CR7] Anderson KA, Anderson DE (1976). Psychologists and spanking. Journal of Clinical Child Psychology.

[CR8] Benjet C, Kazdin AE (2003). Spanking children: The controversies, findings, and new directions. Clinical Psychology Review.

[CR9] Bitensky SH (2006). Corporal punishment of children: A human rights violation.

[CR10] Bussmann, K. D., Erthal, C., & Schroth, A. (2011. Effects of banning corporal punishment in Europe—a five-nation comparison. JE Durrant, Smith, Anne (Hg.): Global Pathways to Abolishing Physical Punishment, 299–322.

[CR11] Child Abuse Prevention and Treatment Reauthorization Act. (2010). Public Law 111–320, 42 U.S.C. 5101 et seq.; 42 U.S.C. 5116 et seq.

[CR12] Committee on the Rights of the Child. (2006). General Comment No. 8 (2006): The right of the child to protection from corporal punishment and or cruel or degrading forms of punishment (articles 1, 28(2), and 37, inter alia) (CRC/C/GC/8). Geneva: United Nations.

[CR13] Durrant JE (2000). Trends in youth crime and well-being since the abolition of corporal punishment in Sweden. Youth Society.

[CR14] Durrant JE, Ensom R (2017). Twenty-five years of physical punishment research: what have we learned?. Journal of the Korean Academy of Child and Adolescent Psychiatry.

[CR15] Durrant JE, Rose-Krasnor L, Broberg AG (2003). Physical punishment and maternal beliefs in Sweden and Canada. Journal of Comparative Family Studies.

[CR16] Ellison, C. G., & Sherkat, D. E. (1993). Conservative Protestantism and support for corporal punishment. *American Sociological Review*, *58*(1), 131–144.

[CR17] Ferguson CJ (2013). Spanking, corporal punishment and negative long-term outcomes: A meta-analytic review of longitudinal studies. Clinical Psychology Review.

[CR18] Gershoff ET (2002). Corporal punishment by parents and associated child behaviors and experiences: A meta-analytic and theoretical review. Psychological Bulletin.

[CR19] Gershoff ET, Bitensky SH (2007). The case against corporal punishment of children: Converging evidence from social science research and international human rights law and implications for U.S. public policy. Psychology, Public Policy, and Law.

[CR20] Gershoff ET, Font SA (2016). Corporal punishment in U.S. public schools: Prevalence, disparities in use, and status in state and federal policy. SRCD Social Policy Report.

[CR21] Gershoff ET, Font SA, Taylor CA, Foster RH, Garza AB, Olson-Dorff D (2016). Medical center staff attitudes about spanking. Child Abuse & Neglect.

[CR22] Gershoff ET, Grogan-Kaylor A (2016). Corporal punishment by parents and its consequences for children: Old controversies and new meta-analyses. Journal of Family Psychology.

[CR23] Gershoff ET, Lansford JE, Sexton HR, Davis-Kean P, Sameroff AJ (2012). Longitudinal links between spanking and children’s externalizing behaviors in a national sample of White, Black, Hispanic, and Asian American families. Child Development.

[CR24] Gershoff ET, Lee SJ, Durrant JE (2017). Promising intervention strategies to reduce parents’ use of physical punishment. Child Abuse & Neglect.

[CR25] Gershoff, E. T., Sattler, K. M., & Ansari, A. (2017). Strengthening causal estimates for links between spanking and children’s externalizing behavior problems. *Psychological Science*. 10.1177/0956797617729816. 10.1177/0956797617729816 PMC5771997 29106806

[CR26] Global Initiative to End All Corporal Punishment of Children. (2017). States which have prohibited all corporal punishment. Retrieved from https://endcorporalpunishment.org/

[CR27] Graziano AM, Namaste KA (1990). Parental use of physical force in child discipline: A survey of 679 college students. Journal of Interpersonal Violence.

[CR28] Gromoske AN, Maguire-Jack K (2012). Transactional and cascading relations between early spanking and children’s social-emotional development. Journal of Marriage and Family.

[CR29] Hornor G, Bretl D, Chapman E, Chiocca E, Donnell C, Doughty K (2015). Corporal punishment: Evaluation of an intervention by PNPs. Journal of Pediatric Health Care.

[CR30] Kish AM, Newcombe PA (2015). “Smacking never hurt me!”: Identifying myths surrounding the use of corporal punishment. Personality and Individual Differences.

[CR31] Knox M (2010). On hitting children: A review of corporal punishment in the United States. Journal of Pediatric Health Care.

[CR32] Knox M, Burkhart K, Cromly A (2013). Supporting positive parenting in community health centers: The ACT Raising Safe Kids Program. Journal of Community Psychology.

[CR33] Knox M, Burkhart K, Howe T (2011). Effects of the ACT Raising Safe Kids parenting program on children’s externalizing problems. Family Relations.

[CR34] Lane LL (1998). The parental rights movement. University of Colorado Law Review.

[CR35] Lansford JE, Cappa C, Putnick DL, Bornstein MH, Deater-Deckard K, Bradley RH (2017). Change over time in parents’ beliefs about and reported use of corporal punishment in eight countries with and without legal bans. Child Abuse & Neglect.

[CR36] Larzelere RE, Kuhn BR (2005). Comparing child outcomes of physical punishment and alternative disciplinary tactics: A meta-analysis. Clinical Child and Family Psychology Review.

[CR37] MacMillan HL, Mikton CR (2017). Moving research beyond the spanking debate. Child Abuse & Neglect.

[CR38] Miller-Perrin C, Perrin R (2017). Changing attitudes about spanking among conservative Christians using interventions that focus on empirical research evidence and alternative biblical interpretations. Child Abuse & Neglect.

[CR39] Moya-Smith, S. (2013). Court: mom who spanked daughter with wooden spoon should not have been labeled child abuser. Retrieved from: https://www.nbcnews.com/news/us-news/court-mom-who-spanked-daughter-wooden-spoon-should-not-have-flna8C11367179

[CR40] National Association of Pediatric Nurse Practitioners (2011). NAPNAP position statement on corporal punishment. *Journal of Pediatric Health Care*, *25*, e31–e32 Retrieved from:https://www.jpedhc.org/article/S0891-5245(06)00410-X/fulltext?code=ymph-site. 22128456

[CR41] Pagliocca, P. M., Melton, G. B., Lyons Jr, P. M., & Weisz, V. (2002). Parenting and the law. In M. H. Bornstein (Ed.), Handbook of parenting volume 5 (pp. 463–485). Mahwah: Lawrence Erlbaum Associates, Publishers.

[CR42] Perrin, R., Miller-Perrin, C., & Song, J. (2017). Changing attitudes about spanking using alternative biblical interpretations. *International Journal of Behavioral Development*, *41*(4), 1–9.

[CR43] Portwood SG, Lambert RG, Abrams LP, Nelson EB (2011). An evaluation of the adults and children together (ACT) against violence parents raising safe kids program. Journal of Primary Prevention.

[CR44] Rae WA, Worchel FF (1991). Ethical beliefs and behaviors of pediatric psychologists: A survey. Journal of Pediatric Psychology.

[CR45] Ruck MD, Keating DP, Saewyc EM, Earls F, Ben-Arieh A (2016). The United Nations Convention on the Rights of the Child: Its relevance for adolescents. Journal of Research on Adolescence.

[CR46] Schenck ER, Lyman RD, Bodin SD (2000). Ethical beliefs, attitudes, and professional practices of psychologists regarding parental use of corporal punishment: A survey. Children’s Services Social Policy Research and Practice.

[CR47] Schworm, P. (2015). SJC affirms parental right to discipline their children. Retrieved from: https://www.bostonglobe.com/metro/2015/06/25/mass-high-court-outlines-legal-rules-spanking/AA75Y9oVRkEBGWIXCoY2fO/story.html

[CR48] Shulman, J. (2014). Does the Constitution protect a fundamental right to parent? Constitution Daily. Retrieved from: https://constitutioncenter.org/blog/does-the-constitution-protect-a-fundamental-right-to-parent.

[CR49] Smith, T. W., Hout, M., Marsden, P. V., & Kim, J. (2015). General Social Survey, 1972-2014. Storrs, CT: Roper Center for Public Opinion Research, University of Connecticut/Ann Arbor, MI: Inter-university Consortium for Political and Social Research [distributors]. https://www.gss.norc.org/.

[CR50] Steinmetz SK, Sussman MB, Steinmetz SK (1987). Family violence: Past, present, and future. Handbook of marriage and the family.

[CR51] Straus MA (2010). Prevalence, societal causes, and trends in corporal punishment by parents in world perspective. Law and Contemporary Problems.

[CR52] Straus MA, Douglas EM, Medeiros RA (2014). The primordial violence: Spanking children, psychological development, violence, and crime.

[CR53] Taylor CA, Fleckman JM, Lee SJ (2017). Attitudes, beliefs, and perceived norms about corporal punishment and related training needs among members of the “American Professional Society on the Abuse of Children”. Child Abuse & Neglect.

[CR54] United Nations (2006). World report on violence against children.

[CR55] United Nations. (2015). UN lauds Somalia as country ratifies landmark children’s rights treaty. Retrieved from: https://news.un.org/en/story/2015/01/488692-un-lauds-somalia-country-ratifies-landmark-childrens-rights-treaty.

[CR56] United Nations, Committee on the Rights of the Child (CRC). (2007). CRC General Comment No. 8 (2006): The Right of the Child to Protection from Corporal Punishment and Other Cruel or Degrading forms of Punishment (U.N. CRC/C/GC/8). Retrieved from: https://www.refworld.org/docid/460bc7772.html

[CR57] United Nations Convention on the Rights of the Child. (1989). G. A. Res. 44/25, U. N. GAOR, 44th Sess., at 3, U. N. Doc. A/RES/44/25. Retrieved from: https://www.unicef.org/crc/

[CR58] Zolotor AJ, Puzia ME (2010). Bans against corporal punishment: A systematic review of the laws, changes in attitudes and behaviours. Child Abuse Review.

